# Assessing the Need and Demand for a Community Emergency Paramedic Strategy in the Ambulance Rescue System of Hamburg, Germany

**DOI:** 10.3390/healthcare13090979

**Published:** 2025-04-23

**Authors:** Marion Sabine Rauner, Benjamin Swyter, Stefan Velev

**Affiliations:** 1 Department of Business Decisions and Analytics, Faculty of Business, Economics, and Statistics, University of Vienna, Oskar-Morgen-Stern-Platz 1, A-1090 Vienna, Austria; 2 Fire Brigade Hamburg, D-20099 Hamburg, Germany; benjamin.swyter@luebeck.de; 3 International Institute for Applied Systems Analysis (IIASA), A-2361 Laxenburg, Austria; velev@iiasa.ac.at

**Keywords:** community emergency paramedics, ambulance rescue system, statistical analysis, manpower planning, resource allocation

## Abstract

**Background:** Demand for Hamburg’s ambulance rescue system (ARS) in Germany, which is managed by the fire service, increased by more than 10% between 2019 and 2021. This increase was mainly driven by a more than 20% increase in non-critical ambulance rescues, while critical rescues decreased over the same period. Factors contributing to this trend include demographic changes, longer waiting times in primary care and declining quality in out-of-hospital care. To address this issue, the introduction of community emergency paramedics (CEPs)—who provide treatment and advice to patients at home before ambulance services are called—has been proposed as a potential solution to alleviate pressure on the ARS. **Methods:** In this study, 17 ARS stations in Hamburg, categorized into three operational areas (East, South, West), were analyzed using comprehensive statistical methods such as hypothesis testing, correlation analysis, regression modeling and clustering. Data from 2019 and 2021 were examined to assess the feasibility of integrating CEPs into the existing system. **Results:** Key findings identified specific stations with high potential for CEP support and optimal mission times (based on time of day, day of week and calendar week) to improve operational efficiency. The impact of regulatory measures introduced during the COVID-19 pandemic was also evident in the 2021 data. **Conclusions:** Finally, four policy scenarios—taking into account different synergy effects among the 17 stations—are presented, providing projections of the managerial and economic benefits for Hamburg policymakers. These policy implications aim to support the development of a robust CEP strategy to improve the overall efficiency and sustainability of the ARS.

## 1. Introduction

Recently, the Severe Acute Respiratory Syndrome Coronavirus (SARS-CoV-2) has hit the world, severely straining health systems and human resources worldwide [1]. Improving the resilience of health systems has therefore risen dramatically on the agenda [1,2,3,4,5,6].

As an example, the demand for the ambulance rescue system (for brevity, we will use ARS throughout the paper) of the City of Hamburg, Germany, which is executed by the fire brigade, rose by more than 10% from 2019 to 2021 [7]. This increase in demand can be mainly attributed to the increase in the number of non-time-critical ambulance rescue operations by more than 20%, while the number of critical ambulance rescue service operations even dropped. This trend is caused by demographic changes, long waiting times in primary care, and the deterioration of the extramural healthcare services, as well as the COVID-19 (Coronavirus Disease) pandemic. To cope with this situation, fire brigade staff have often taken over the tasks of the ARS’s duties since then.

### 1.1. The Ambulance Rescue System in the City of Hamburg, Germany

Following the Second World War, the responsibility for the ambulance rescue service was transferred to the fire brigade in the City of Hamburg [8], which runs 17 large rescue stations organized into three directorates located in the East, West and South, as outlined in Section 2.1. Prior to this, the fire brigade did not undertake such duties. To supplement the larger fire and rescue stations, there are smaller rescue stations in Hamburg that address the issue of response times, although these are not included in our study. There are a total of 26 smaller locations, distributed differently across the city. In some cases, they have only a single vehicle or just a few emergency vehicles that are assigned to one of the three directorates; not all of those stations are staffed by the fire brigade, and not all are operational on a 24/7 basis.

In the event of an emergency, callers should dial 116 117 for minor cases or 112 for more serious issues [9]. For minor cases, callers will be directed to a health insurance emergency service. For more serious issues, callers will be directed to the rescue coordination center of the fire brigade in the City of Hamburg. The health insurance emergency service provides medical assistance outside the standard hours of medical doctors with private offices. Their medical staff can assist with identifying nearby medical practices and pharmacies, arranging doctor visits and scheduling appointments. For more severe cases, they will refer the caller to the hotline 112, which is staffed by trained firefighters who are at least all paramedics. The “call taker” can provide first aid instructions or instruct births or resuscitation via the telephone. The dispatcher’s role is to alert and direct vehicles to the appropriate location. The “standardized emergency call query” DIASweb from Noratec is used to process and assess emergency calls, generating an alarm keyword via a query algorithm with predefined questions, answer options and results. This alarm keyword is passed to the dispatcher, who then assigns vehicles. If necessary, the call taker remains on the line with the caller. Vehicles are selected via routing by the operations control computer or manually by the dispatcher.

### 1.2. The General Potential of CEP Systems

With an emergent shortage of health professionals, long ambulance journey times and a lack of sites for new facilities, the conventional strategy for meeting the increasing demand for pre-hospital emergency care by providing additional human and material resources is no longer sustainable [7]. An innovative potential solution to alleviate this overburdening situation could be the introduction of community emergency paramedics (CEPs) to treat and advise non-severe injured patients at home, before the ambulance service might be needed [10,11,12,13,14,15]. Past reviews have shown that the CEP systems can additionally increase patient health outcomes and satisfaction, better reach disadvantaged communities, enhance provider satisfaction and reduce emergency department and inpatient utilization, such as in the United States of America [16] and Western nations [17]. While worldwide CEP systems vary in terms of their structure and the distribution of competencies among medical staff, the efficiency of intramural and extramural emergency care and healthcare systems overall can be enhanced.

In Germany, CEPs are trained at the highest advanced level (Notfallsanitäter), i.e., advanced paramedics, below physicians [7]. CEPs have various treatment competencies, such as changing urinary catheters or administering follow-up medications, but not certain competencies such as medical prescription, diagnosis, or performing surgeries. One CEP takes a small vehicle and emergency equipment without further staff present, differing from regular ambulances, which carry, at least one general and one advanced paramedic to visit one non-severely injured patient. If a non-emergency call subsequently results in an emergency call, the CEP will summon a more advanced ambulance to attend to the patient, who might then be transported after initial treatment to a hospital [18]. More advanced vehicles (emergency medical cars, emergency medical vehicles, emergency transport vehicles, intensive care transport vehicles) are typically equipped with at least one physician and at least one general paramedic (emergency medical cars), one general and one experienced general paramedic (emergency transport vehicles), as well as one general and advanced paramedic (emergency medical vehicles) for the treatment (all of the abovementioned vehicles) and transport (except emergency medical cars) of severely injured patients, respectively. However, it should be noted that emergency helicopters are operated by a dedicated flight crew, which is not usually part of the medical treatment team and includes at least two advanced paramedics. Consequently, a CEP strategy would liberate both staff and emergency vehicles for the management of non-severely injured patients.

To assess the potential for the suitability of the application of CEPs to the ARS of the City of Hamburg, Germany, for about 1.9 million inhabitants, we focused on countries with comparable legal foundations and similar structures of rescue systems, such as Austria. In addition to counselling emergency calls, the lower Austrian CEP system acts as a first responder to bridge long waiting times until the arrival of the emergency services [19,20]. During a test phase from May 2020 to December 2022, 5367 operations were documented. In 60% of the operations, nursing was required, while 40% of the operations needed emergency care, with a rate of 38% of patients not transported to hospitals.

In Germany, pioneer pilot studies can be found, for example, in the cities of Frankfurt (about 773.000 inhabitants) [21], Bremen (about 569.000 inhabitants) [22], Braunschweig (about 251.000 inhabitants) [23], and Oldenburg (about 172.000 inhabitants) [10]. The ambitious aim of the related policymakers is to implement and finance CEPs in those cities in the upcoming years.

In a pilot study in the train station area of Frankfurt, 176 operations were executed by CEPs in fall 2022 [21]. In 53.4% of all operations, the resource was alerted alone, and in 46.6%, it acted as a first responder in addition to the ordinary resource of the rescue service (ambulance or emergency ambulance vehicles). Only in 14.2% of the independent operations was the rescue service additionally requested.

A similar success was reported in Bremen, where the CEP concept was first introduced for visiting COVID-19 patients at home in March 2020 and afterwards applied to study the potential for non-critical emergency calls [22]. From the about 2500 operations, 72% of the patients needed no transportation to hospitals, and only 14% of the patients additionally required the ambulance rescue service. Due to this success, the CEP system—called “HanseSani”—was introduced in Bremen.

Seeger et al. [10] reported in a pilot study in Oldenburg and its surroundings that 59% of CEP operations needed no transportation to hospitals in 2019, while Günther et al. [23] found that 65% of general ambulant emergency cases needed no transportation to hospitals.

To summarize, CEP systems have the capacity to release ARS; however, it is important for policymakers to acknowledge that the increasing demand is also driven by the treatment of patients outside business hours in the context of contractual medical care [24], which is not the primary intention of an ARS. For instance, Seeger et al. [25] found that, in a survey of over 5000 German patients, many patients attempted to contact the rescue services, despite these services not being the intended purpose of the ARS. The solution to this access problem lies in the extension of the opening hours of contractual medical care, particularly the concept of primary healthcare centers, the enhancement of emergency hotlines and the introduction of community nurses. The CEP system also integrates well with modern app-based first-responder alert systems and publicly located automated external defibrillators, further reducing response times for first aid [26]. The final aim is a mobile-integrated healthcare, a patient-centered coordination of a team led by a physician and including essential healthcare professionals (e.g., CEPs, community nurses, social/mental healthcare workers, family doctors, other medical specialists) [27].

### 1.3. The Potential of a CEP System for the City of Hamburg, Germany

Despite the growing interest in CEPs, significant research gaps remain. For example, it is unclear how non-time-critical call volumes vary across different urban areas and which staffing strategies might be most beneficial for a particular area or city. To address these research gaps, we have formulated the following research questions for our need and demand case study of the City of Hamburg, Germany:

1. Can a station-level analysis for the 17 major rescue stations identify areas with the greatest potential for non-time-critical CEP interventions?
2. Which time-of-day or seasonal factors significantly influence non-critical call patterns?
3. How could these insights inform policymakers about resource allocation?
4. Does an uneven distribution of call volume (high-demand vs. low-demand areas) require tailored CEP strategies, especially at peak times?

We demonstrate that the ARS could be released by the introducing a CEP system for past data from 2019 and 2021, which could be essential for policymakers.

Section 2 describes the ARS of the City of Hamburg, the data used and the methods applied. We then present the results for the 17 major rescue stations and their regional operations (areas: East, South and West) of the ARS, which are analyzed in detail using statistical analysis with DataTab Version 2024, R 4.4.2 and IBM SPSS Statistics 29.0.1, such as hypothesis testing, correlation analysis, regression models and clustering, to identify the potential for a sound area- and time-related CEP strategy in Section 3. In Section 4, we identify specific stations with high potential, as well as the best times (time of day, day of week, calendar week) for supporting them with CEPs. Section 5 concludes the paper with policy implications and further research.

## 2. Materials and Methods

### 2.1. Purpose of the Case Study

**Figure 1:**
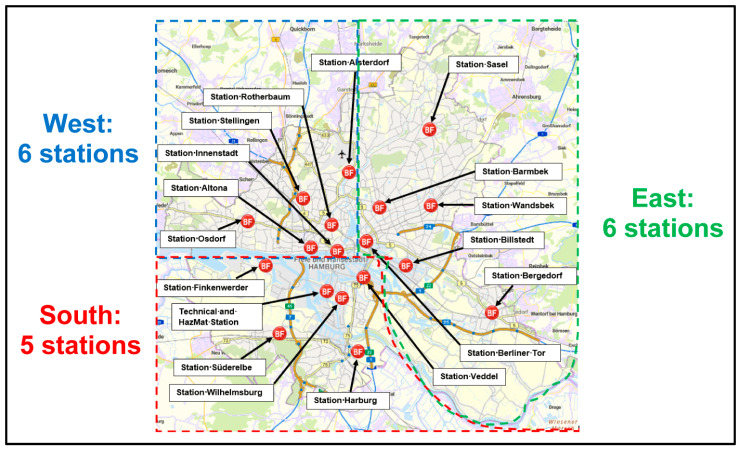
The area map of the 17 major ARS stations in the city of Hamburg, Germany, 2019.

The purpose of this need and demand case study “for the city of Hamburg, Germany” was to investigate the potential of the introduction of CEPs, who would treat and counsel non-severely injured patients—at home or in public—that did not require subsequent transportation to hospital [7]. We therefore focused on non-time-critical, routine ambulance services provided by the 17 major ambulance stations in the East (six stations), South (five stations) and West (six stations, including the city center) areas shown in Figure 1. Excluded from this were special operations, such as police special operations, or other resources used for technical assistance or firefighting. All ambulances were allocated to stations, except for two which were assigned to area directorates in 2021 and 2022 [7].

We aimed to identify certain high-potential stations and the best times (time of day, day of the week, calendar week) for them to be supported by CEPs. We thus collected data on the number and type of operations, calendar week, day of the week and time of day for non-time-critical calls, which included both ordinary non-event and ordinary event emergency calls with and without patient transport to hospital. Data were collected for the year 2019, to exclude the COVID-19 effect, and for the years after 2020, when the situation was more normalized. It was also reported in the literature that the quality of medical services was highly affected, especially early in the COVID-19 pandemic (see, e.g., [28]).

Our investigation can be used as a starting point for a detailed simulation study prior to real implementation or as a guideline for a pilot study in the entire city or in selected stations using more detailed current data. Including the 26 small rescue stations, considering “neighborhood and overland support” in case of overflow of the systems, reallocating large and small rescue stations and/or related vehicles, or routing strategies of vehicles depending on the traffic and hospital locations are subjects of a huge potential future research project.

Finally, policymakers need to consider that CEPs have a limited scope, mainly for non-time-critical and less severe patients, as described above. For severe and mostly time-critical medical cases, more emergency medical personnel and advanced equipment and vehicles are needed.

### 2.2. Data of the Case Study

The data used in this system originate from the operations control system of the Hamburg Fire Department’s rescue coordination center [7]. The system generates a data record for every call received via the emergency hotline, 112, or manually entered emergency operations, which are then stored in a dedicated database. Known as “HELS HamburgerEinsatzLeitSystem”, this system is used jointly by the Hamburg Fire Brigade and the Hamburg Police. It is characterized by its monolithic structure and localized operation, with no external server storage for data.

From this database [7], we obtained anonymous data in an Excel spreadsheet for further analysis. One of the authors has worked at the Hamburg Fire Brigade for many years, including during the main part of the study, which is why we obtained full-year data for 2019 and 2021. For each patient, the ambulance type sent is recorded, from which one can derive whether the call was classified by the call center as time-critical or non-time-critical during triage (cf. Section 1.2). We focused on non-time-critical patients to whom an ordinary ambulance car and related staff without the use of special rights and rights of way is sent, as these missions could be executed by CEPs. Due to the extraordinary effects of the COVID-19 pandemic, including strict lockdowns and many emergency measures in 2020, we excluded data from that year to avoid biasing the analysis. We also partially compared 2021 data with 2022 data. We did not collect data for 2023 because the first few months of the year were still affected by the COVD-19 pandemic, and the ARS policymakers decided not to introduce a CEP system.

The data obtained covered all ambulance rescue operations, including details such as the type of incident, location, time and patient outcome. Prior to analysis, all data were fully anonymized. Access to the data was granted by the Hamburg Fire Brigade under a data-sharing agreement in order to ensure compliance with privacy regulations.

### 2.3. Statistical and Policy Analyses Performed

For our statistical analysis, we focused on the dataset for 2019 by analyzing different patterns of non-critical ambulance rescues performed by the 17 stations over 365 days, 52 weeks (12 months), seven days/week and 24 h/day, as shown by the associated variables used in Table 1. We also examined the effect of holidays and events during the weeks of the year. The following longer holidays were taken into account (variable ‘breaks’): (1) ski holidays, (2) Pentecost, (3) summer holidays, (4) autumn holidays and (5) Christmas. In addition, all legal holidays (variable “holidays”) of the City of Hamburg (e.g., New Year, Easter, Labor Day) were taken into consideration. Finally, all major events were included (variable “events”): (1) spring, summer and winter fairs, known as “DOM”, (2) Harley Days, (3) Schlagermove, (4) Christopher Street Day, (5) Harbour Birthday and (6) Christmas market season.

We performed several statistical analyses to investigate the data in detail using DataTab, R and SPSS. First, we used hypothesis tests, specifically Kruskal–Wallis [29] (a non-parametric test was chosen due to normality concerns), to pinpoint statistically significant differences among stations, weeks, months, days of the week and hours of the day, followed by Dunn–Bonferroni [30,31] post hoc comparisons where necessary (using DataTab and SPSS) in Section 3.1.1, Section 3.1.2, Section 3.1.3 and Section 3.1.4 [7]. However, diagnostic checks (Durbin–Watson statistics [32,33], residual plots) sometimes revealed significant autocorrelation, violating linear model assumptions (using DataTab and SPSS) for our initial linear regression models to investigate time-related patterns. Consequently, we shifted to a Spearman’s rank correlation approach [34] to capture monotonic relationships without requiring normality or independence. We therefore employed Spearman’s correlation to assess relationships between time-related variables (e.g., day of the week) and call volume and incorporated regression models to explore potential linear or non-linear trends (using DataTab and SPSS).

Furthermore, we identified stations with the highest number of non-time-critical ambulance operations by applying hierarchical clustering [35] (using average linkage and Euclidean distance in DataTab) in Section 3.1.1, thereby providing insights into resource allocation strategies among stations. As a final check, we also tested the validity of our results using a Bayesian additive regression model with a piecewise linear trend and Fourier-based seasonalities, commonly referred to as the Prophet model [36], by using R in Section 3.1.5 (extension of [7]). We evaluated their robustness using a cross-validation methodology and posterior credible intervals to ensure that they were robust. Then, given that we had already applied a Fourier decomposition [37] of the time series data for the Prophet model, we used it to also identify cyclical patterns across different time scales (yearly, monthly, weekly and daily), while also controlling for workdays, holidays, school breaks and events. By decomposing the aggregated demand curves into sinusoids, we were able to isolate both seasonal trends (e.g., annual, monthly and weekly) and short-term fluctuations (e.g., daily peaks). Additionally, we assessed the most suitable operating times during the day for CEPs by a need and demand analysis of each of the 17 stations in Section 3.2. Finally, three different policy strategies for allocating CEPs among the stations are presented based on the total number of working hours per year.

## 3. Results

In investigating the best strategy for implementing the CEP concept in the city of Hamburg, we first performed a descriptive analysis of the number of non-time-critical ambulance operations, including statistical tests, clustering methods and regression models (Section 3.1), to find trends and patterns in the data. In Section 3.2, we analyze three practice-oriented strategies to best implement the CEP concept, taking into account the results of our statistical investigations depending on key internal and external environmental conditions.

### 3.1. Descriptive Analysis Including Statistical Tests, Clustering Methods and Regression Models

We analyzed the results regarding patterns in the dataset for non-critical ambulance operations in the City of Hamburg regarding area effects (Section 3.1.1), weekly effects (Section 3.1.2), weekday effects (Section 3.1.3), time of the day effects (Section 3.1.4), and time series effects (Section 3.1.5), and summarized all area- and time-related effects found (Section 3.1.5).

#### 3.1.1. Area and Station Effects

An initial investigation was conducted of the differences in population density (see Table 2) and the number of operations performed (see Table 3) in the City of Hamburg, Germany, according to area and station [7]. The stations are allocated to the three areas (East, South, West) with huge areas above 50 km^2^ and below 100 km^2^ (e.g., Billstedt or Sasel in the East area above the Elbe River; Finkenwerder or Süderelbe in the South area; Osdorf in the West area). Population density and operational metrics were analyzed across the three areas (East, South, West), with the West area experiencing the highest concentrations of events (e.g., DOM, Schlagermove, Harbour’s Birthday). The West area is home to a significant proportion of the city’s population, with some areas, such as Osdorf and Stellingen, having over 200,000 residents. Similarly, the East area also has a population of over 100,000. The South area has the lowest population density.

The five stations with the highest population density are as follows: (1) Rotherbaum, (2) Barmbek, (3) Altona, (4) Berliner Tor and (5) Stellingen. The mean population density was 2401.10/km^2^ for the City of Hamburg, while the mean population density amounted to 5336.39/km^2^, 3190.87/km^2^ and 1276.29/km^2^ in the West, East and South areas, respectively.

A Kruskal–Wallis test was used to statistically investigate area differences, revealing a significant population density difference among the three areas (*p* = 0.023). The Dunn–Bonferroni test showed that the South area and the West area significantly differed from each other (adjusted *p* = 0.018), making this result important for different area strategies.

As shown in Table 3 [7], CEPs were able to execute 48,035 non-time-critical operations. The West area, which is heavily populated, the East area, which is densely populated, and the South area, which is sparsely populated, saw 22,696, 18,148 and 7191 of such operations, respectively (a total of 48,035, or 16.4% of overall operations). The average number of operations amounted to 2825.59 per station, ranging from Finkenwerder executing only 383 to Wandsbek performing 4883 (italic lines in Table 3). This reveals the potential for applying the CEP strategy—especially in certain areas and stations—which will be investigated in more detail.

Firstly, we investigated differences in the number of non-time-critical operations performed in the three areas using Kruskal–Wallis and Dunn–Bonferroni post hoc tests. We found statistically significant differences in the population densities of the areas and investigated these findings for the operations performed in three areas (East, South and West), totaling 48,035 operations performed by 17 stations (*p* < 0.012). However, further analysis revealed additional variations among the areas: The East area exhibited significant disparities compared to the South area (adjusted *p* = 0.011), yet not in relation to the West area (adjusted *p* = 0.109). The same pattern was also statistically confirmed for the number of operations with transportation to hospital (*p* < 0.017) and without transportation to hospital (*p* = 0.004), with a significant difference only among the East and South areas with an adjusted *p* = 0.016 and *p*= 0.003, respectively. Thus, differences among some areas were found.

Next, we analyzed whether the total number of non-time-critical operations correlates to the number of non-time-critical operations that did not result in hospital transportation. A subsequent Spearman correlation revealed a statistically significant, very high positive correlation (*r*(15) = 0.90, *p* = 0.001), confirming that a similar proportion of patients would not be transported to the hospital across the different stations, ranging from 15.20% (Sasel) to 29.89% (Finkenwerder). This information is essential for saving resources, because for those patients not transported to the hospital, no expensive ambulance vehicle and staff are subsequently required, and only one CEP would be sufficient for treatment.

**Figure 2:**
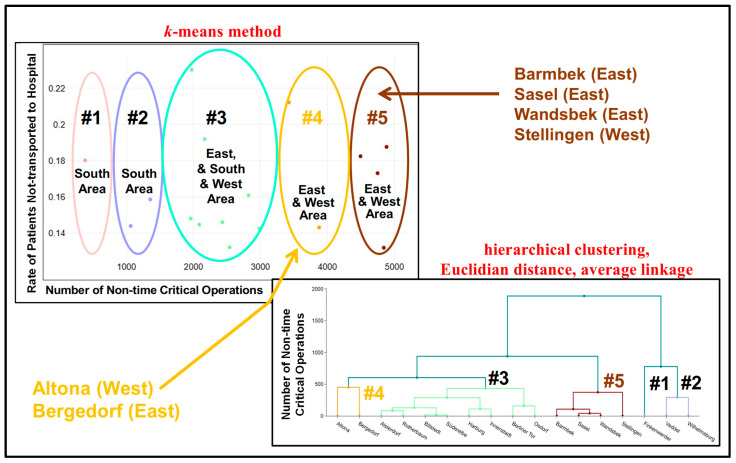
Assignment of the 17 major ARS stations to five clusters in the City of Hamburg, Germany, 2019.

Finally, we disclosed high potential areas for applying the CEP strategy, along with their related stations, by using hierarchical clustering techniques. These techniques allowed for five clusters, as illustrated in Figure 2, based on the data of Table 3 [7].

As illustrated in Figure 2, the four high-demand stations (Barmbek, Sasel, Wandsbek and Stellingen) are allocated to cluster #5 (brown), which has a high number of non-time-critical ambulance operations (above 4000) and a rate of between about 13% and 19% of patients not being transported to the hospital. Cluster #1 (pink) contains the low-demand station Finkenwerder (South area) and cluster #2 (lila) includes Veddel and Wilhelmsburg (South area), which would be poor candidates for introducing a CEP strategy. The other stations are assigned to cluster #3 (green), except the stations Altona and Bergedorf, which form cluster #4 (orange). If only four clusters were to be permitted, cluster #4 would be merged into cluster #3.

#### 3.1.2. Weekly Effects

**Figure 3:**
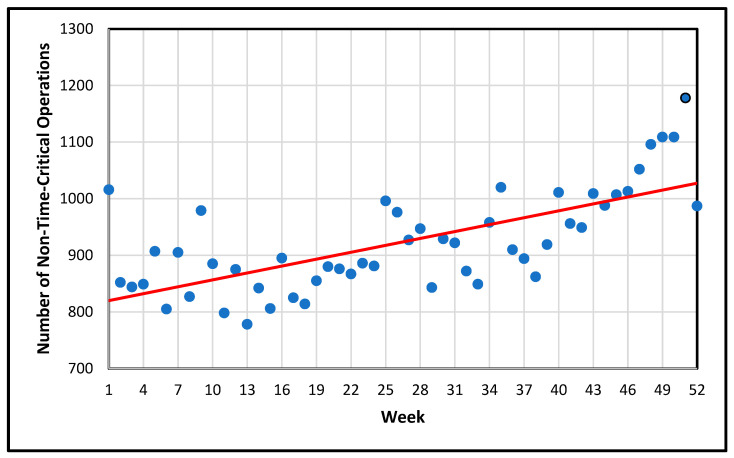
Positive correlation between the week and the number of non-time-critical operations performed by the 17 major ARS stations in the City of Hamburg, Germany, 2019.

An assessment was made of seasonal weekly effects for the number of non-critical operations performed in 2019 (cf. Figure 3) [7]. From the beginning of the year, the number of operations increased slightly from 1016 due to higher temperatures, and then from autumn onwards due to the Advent markets, which began in weeks 47/48 and continued until week 51. The average number of operations was 923.75 per week. The outliers represented either the weeks of school holidays (e.g., skiing break, summer break, fall break), event weeks (e.g., Advent season), or the last week of the year between Christmas and New Year, when the operations dropped down below 1000—around the level before the Advent season.

Linear regression for the number of non-time-critical operations depending on the week did not meet the “non-autocorrelation” criterion of the residual (Durbin–Watson-test: *p* = 0.003, which should be above 0.05). Consequently, we proceeded to test for a positive correlation using the Spearman correlation method, which yielded a high positive correlation coefficient (*r* = 0.67, *p* < 0.001), thereby confirming the above findings. In order to investigate different non-linear time-related effects, the Prophet model [36] was used in Section 3.1.5.

#### 3.1.3. Weekday Effects

As shown in Table 4 [7], the number of operations performed each day ranged from 6599 to 7125, with an average for 6862.14 per day.

Utilizing a linear regression model, only 17% of the variance in operations could be explained (*p* = 0.341; *R*^2^ = 0.17; adjusted *R*^2^ = 0.01) among the days of the week. A moderate, negative correlation was obtained, which was not statistically significant, using a Spearman correlation *r*(5) = −0.39 (*p* < 0.383). Consequently, policymakers might not need to consider a weekday effect, as further explored using the Prophet model [36] (cf. Section 3.1.5).

#### 3.1.4. Time of the Day Effects

**Figure 4:**
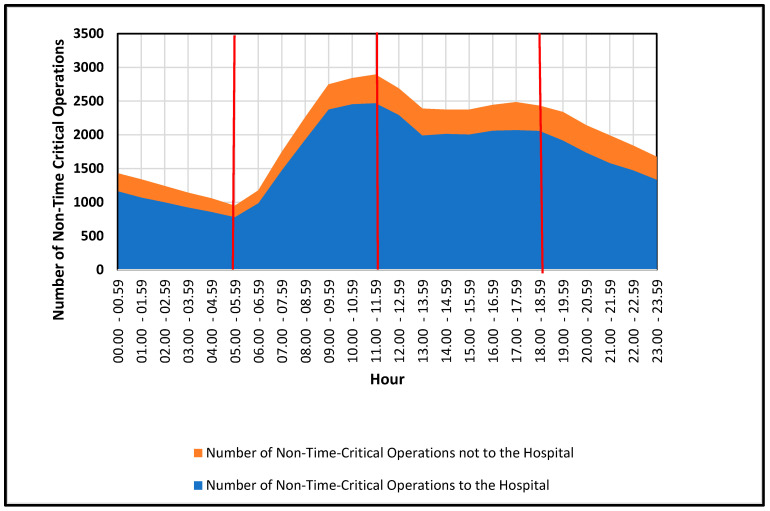
Number of non-time-critical operations with and without transportation to the hospital during the hours of the day in the City of Hamburg, Germany, 2019.

During the day, the demand patterns observed in the dataset were found to be dependent on the activity patterns of people, which were separated into four groups (cf. Figure 4) [7]: (1) night, from 0.00 to 5.59 (low activity: sleeping); (2) morning, from 6.00 to 11.59 (increasing activity: getting up, leaving home, child caring, studying, working); (3) afternoon from 12.00 to 18.59 (high activity with a decreasing trend: e.g., child caring, studying, working, arriving home); and (4) evening, from 19.00 to 23.59 (lower activity with a decreasing trend: e.g., relaxing, sleeping).

The hourly demand pattern was found to be non-linear for the entire day, and this will be further investigated by the Prophet model [36] in the next Section 3.1.5.

#### 3.1.5. Time Series Effects

To validate and refine the temporal patterns identified in Section 3.1.1, Section 3.1.2, Section 3.1.3 and Section 3.1.4, we employed the Prophet model [36]. The Prophet model is a Bayesian additive regression method that integrates piecewise linear trends, Fourier-based multiple seasonalities and holiday/event effects in a single comprehensive framework. We modeled the counts of non-time-critical ambulance calls (including normal days, school breaks, holidays and events) for the year 2019. We then isolated distinct cyclical structures (these being yearly, monthly, weekly and daily) and assessed their relevance using posterior credible intervals and cross-validation.

##### Overview of the Prophet Model

The Prophet model [36] comes from the assumption that the observed time series y(t) can be decomposed into three main deterministic components plus noise:

(1) $$y\left(t\right)=g\left(t\right)+s\left(t\right)+h\left(t\right)+\epsilon_{t}$$

where

- ***g*(*t*), trend:** To capture any trend effects, the Prophet model uses a piecewise linear specification that accommodates “changepoints” (dates when the slope changes), thus capturing gradual or abrupt shifts in call volume over the year. For the most part, there is no long-term increase or decrease in call volume. The trend is slightly positive, but that is due to the increase in end-of-year seasonality and is therefore non-informative.
- ***s*(*t*), seasonality:** To capture the multiple seasonalities needed for this analysis, the Prophet model utilizes Fourier series. Each series reflects a different cycle: daily (24 h), weekly, monthly and yearly.
- ***h*(*t*), holiday or event effects:** Known holidays, major events and school breaks were entered as time-limited regressors. This allows the Prophet model to estimate specific localized changes in call volumes.
- ϵ***_t_***, **residual error**.

##### Fourier Decomposition of Seasonalities

To handle multiple types of cyclical patterns simultaneously, the Prophet model incorporates a Fourier decomposition [37]. This approach allows us to capture daily, weekly, monthly and annual cycles by expressing each seasonal period P by sine and cosine terms (e.g., P=7 for weekly patterns, P=365.25 for annual):

(2) $$s\left(t\right)=\sum_{k=1}^{K}\left(\alpha_{K}cos\left(\frac{2\pi kt}{P}\right)+\beta_{k}sin\left(\frac{2\pi kt}{P}\right)\right)$$

Here, the K parameter controls how many terms (harmonics) are used, or, in other words, how flexible the model’s seasonality is. Larger K values allow for more intricate (complex) oscillations of the modeled seasonal pattern. The model estimates the amplitude and phase parameters (*α_k_*, *β_k_*) of each sinusoid to best fit the observed patterns (e.g., a 24 h cycle, a 7-day weekly cycle, or a 365.25-day annual cycle).

##### Bayesian Estimation and Posterior Credible Intervals

We use the Hamiltonian Monte Carlo [38] method to fit the Prophet model. This methodology allows us to assign a posterior distribution for each model parameter (e.g., trend slopes, Fourier amplitudes, holiday/event coefficients) based on its likelihood and prior [39]. From these posterior distributions, we can derive credible 95% intervals, which indicate the plausible ranges of the parameter values.

A 95% credible interval for a specific parameter that does not include zero is regarded as “significant”, in the Bayesian sense. For example, a significant yearly amplitude indicates that the yearly cycle’s effect on call volume is consistently non-zero across the posterior samples.

##### (Time Series) Cross-Validation

Having specified the components of the Prophet model, we used time series cross-validation (CV) to determine the out-of-sample performance of the model. We also used CV to validate the results from the posterior credible intervals by comparing models with differing components (e.g., to test for differences in predictive performance between a model with and a model without a yearly trend component). The CV results agree fully with the posterior credible interval assessment. Unlike CV for independent data, time series CV must respect chronology. Here, we use a sliding window approach using the following steps:

1. Train on data up to a cut-off date.
2. Forecast the next horizon (e.g., 14 or 30 days).
3. Compare predictions to actual values using the RMSE (root mean squared error).
4. Roll the cut-off forward and repeat.

By aggregating the errors over multiple windows, we ensure that our seasonalities and holiday/event effects are generalizable and not over-fitted to short-term shocks or anomalies.

###### Summary of the Area- and Time-Related Effects Found

**Figure 5:**
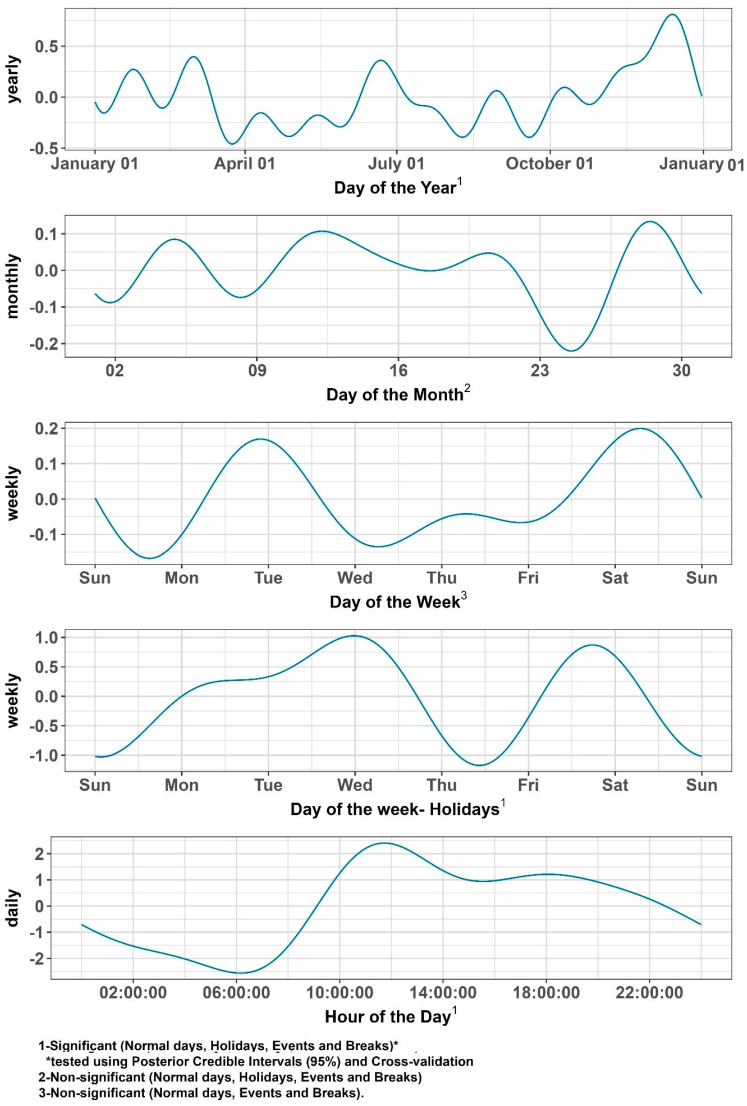
Time series cross-validation (day of the year, day of the month, day of the week and hour of the day) for the number of non-time-critical operations in the City of Hamburg, Germany, 2019.

From our Prophet-based Fourier decomposition (see Figure 5), we extracted four main cycles regarding seasonality. We tested each cycle under different “treatments” of the data (normal days, breaks, holidays, events) using both their 95% posterior credible intervals and CV. Here is what we observed for the policymaking illustrated in Figure 5:

1. **Yearly Seasonality:** Informative and “significant” for all data subsets (normal days, holidays, events, school breaks). All 95% posterior credible intervals show an effect different from 0 and including them gives a much better performance when using cross-validation compared to the alternative model (one without). This shows a robust yearly pattern, with higher volumes typically around the semester break, mid-summer and pre-Christmas.
2. **Monthly Seasonality:** Not informative under any treatment. The posterior intervals included zero, offering no consistent monthly effect. Including a monthly effect did not increase the model’s performance in terms of RMSE.
3. **Weekly Seasonality:** Informative only when we explicitly model weeks with holidays. For normal days, breaks and event weeks, the weekly pattern was non-informative (credible intervals included zero, and the performance did not increase when doing CV).
4. **Daily Seasonality (24 h cycle):** Informative across all categories, showing a pronounced morning-to-afternoon peak and lower overnight volumes. When comparing different treatments, the main difference among holidays, events and breaks compared to normal days is that the trend is slightly shifted left or right, which indicates an on average earlier or later start of day. The overall shape of the trend, however, remains the same.

When we analyzed the 2021 dataset, we found that major restrictions on business openings and closures (and a reduced frequency of Advent markets) or contact restrictions had led to a reduction in operations to curb the effects of the COVID-19 pandemic [7]. However, due to the impact of the disease, there was an increase in overall operations in the less populated East and South areas by about 19% and 22% compared to 2019, respectively, while those operations only increased by about 14% in the highly populated West area compared to 2019. As the demand for services has risen, a post-COVID-19 dataset [40] for 2024 or onwards would be ideal for further analysis. Some of the increase in demand is caused by the fact that COVID-19 has become prevalent and long COVID-19 effects have manifested in the population as well as in the workforce, which might increase the demand for ambulance services, and thus, CEPs [41,42,43].

In conclusion, for policymakers especially, these hourly time effects are a significant consideration when allocating resources among stations and areas, particularly in regard to peak hours or half-shifts of CEPs. In addition, several stations in the West and East areas are highly suitable candidates for the CEP strategy. For instance, the Wandsbek station in the East area, which conducted 4883 operations, and the stations of Barmbek, Sasel and Stellingen in the East area, which conducted over 4000 operations, would be the optimal starting points for the introduction, as outlined in Section 3.2 on the allocation strategies of CEPs among the 17 stations.

### 3.2. Allocation Strategies for the Community Emergency Paramedics Concept in the City of Hamburg

A CEP concept would require additional staff, vehicles, equipment and parking space for vehicles at stations [7,44]. As medical staff is the most crucial and costly element, we focused on the number of additional staff members and their working hours per year as a first step, in addition to the current resources. The other cost components would have been too complex and costly to include in this preliminary analysis and could be the subject of further research in a comprehensive cost-effectiveness analysis [45]. Then, the total running costs of a basic situation would have to be compared to the total running costs of situations including CEPs. This could be done using advanced simulation methods with a comprehensive cost-effectiveness analysis [46,47,48,49,50].

In the emergency services sector, the requirements plan [44] (p. 167) specifies suitable, medically necessary, efficient and economically justifiable reserve capacities for emergency rescue. In addition, the required response time, size and condition of the catchment area, population size and other aspects should be considered. As we deal with non-time-critical ambulance operations, they are labeled as non-emergencies. Ambulance cars do not have any special rights or rights of way. In our policy scenario, one CEP would drive one vehicle for such an operation and would be assigned to one or more predefined stations #1–#17. The idea of free-floating CEPs among stations could be a topic for further research. Thus, an ordinary frequency average calculation can be performed for each of the stations.

As outlined in Section 3.1.5, we found that significant demand patterns emerged during the day and among stations, as well as during certain holiday seasons. Therefore, when engaging with policymaking, the general concept for staff planning for emergency services [25] (p. 73) can be utilized as a foundation. In this instance, the necessary number of vehicles—and, in our case, CEPs—is calculated based on the average frequency rate of operations at the time of day (average number of alerts per hour per year/365 days) multiplied by the required average operating time. In the pilot study of Oldenburg and the surrounding area (3131 km^2^) by Seeger et al. [25], CEPs required approximately 63.3 min per operation. For the city of Hamburg (755 km^2^), a value of 60 min would be an appropriate upper point for a preliminary calculation. Furthermore, a workload of 75–85% per staff member in the ambulance sector would be adequate to allow time for administration, recovery and accounting for differences between weeks. Based on such a calculation, the number of CEPs required per station can be determined.

**Figure 6:**
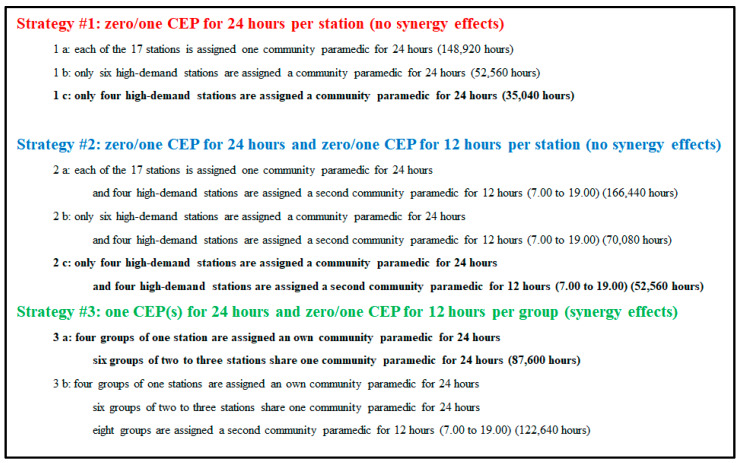
Investigated CEP implementation strategies for the City of Hamburg, Germany, 2019.

Currently, the working hours of the staff members of the fire brigade and ambulance system of the City of Hamburg are 12 h (from 7 a.m. to 7 p.m. or from 7 p.m. to 7 a.m.) or 24 h (from 7 a.m. to 7 a.m.), with 48 working hours per week for civil servants. Therefore, we designed three implementation scenarios for CEP strategies for the policymakers of the City of Hamburg, as shown in Figure 6 (revised based on [7]). CEP strategies #1 [a, b, c] and #2 [a, b, c] would allocate resources to only one station, while in CEP strategy #3 [a, b], geographically nearby low-demand stations would share resources, forming a group. Depending on economic and political reasoning, policymakers would then decide which of the strategies might be most adequate for them, as illustrated in Section 4.

#### 3.2.1. CEP Strategy #1

CEP strategy #1[a] would be an equal distribution of resources among the 17 stations without considering their different demand patterns, as illustrated in Section 3.1. Policymakers would assign one CEP to each of the 17 stations over 365 days for 24 h, which would amount to 17 × 365 × 24 = **148,920 staff hours per year**.

For example, the Wandsbek station performed the highest number of non-time-critical operations, amounting to 4883. If one CEP were provided for the entire day, then the workload per hour would be below 85%, except for the time slot from 10 to 11 a.m. (marked in bold in Table 5, revised based on [7]). However, the estimated average workload was always below 100% for this station. In the time slot from 10 to 11 a.m., the average rate of non-time-critical operations would be about 0.8877, with an assumed average operations time of 60 min amounting to an average operation time demand of about 53.26 min (60 × 0.88). As one CEP was available, the operational readiness time amounted to 1 × 60 = 60 min, with an average workload of 53.26/60 × 100 = 88.77%. In particular, during the night hours from 2 a.m. to 7 a.m., the workload was <35.34% per hour.

For the 17 stations, we found that one CEP for 24 h per day would have an average workload of about 51–56% per hour for the high-demand stations (indicated with “b, c” in Table 6) of Barmbek (East area), Sasel (East area), Stellingen (West area) and Wandsbek (East area) in the pre-COVID-19 dataset for 2019. However, for the Stellingen and Wandsbek stations in 2019, the average workload per day hour was above 85% but below 100% for the time slot from 9.00 to 10.00, as illustrated for the Wandsbek station above. When we analyzed the COVID-19 dataset for 2021, this effect was also true for the two other high-demand stations, Barmbek and Sasel. Thus, all four high-demand stations would be good candidates for the introduction of CEP strategy #2 [a], in which a second CEP would be assigned to each of them from 9.00 to 19.00 to account for a realistic shift and to cover the main peak hours. Finkenwerder is not a realistic candidate for the introduction of a CEP strategy, which was also shown in the cluster analysis.

Table 6 (new based on [7]) illustrates the average hourly workload per station for strategies #1 [a, b, c]. Regarding the other stations of the West area, the average workload per hour varied between about 23.79% (Rotherbaum) and 44.26% (Altona), while the other stations of the East area had higher average workloads per day hour, ranging from 22.51% (Billstedt) to 39.09% (Bergedorf). Policymakers would have to decide which average workload per hour might be acceptable for the introduction of this CEP strategy #1 in the two higher-demand stations of Altona and Bergedorf (indicated with “b” in Table 6).

However, the low-demand stations in the South area would be poor candidates for the introduction of this CEP strategy #1 (indicated with “a” in Table 6). For example, in the low-demand Finkenwerder station, the average workload amounted to 4.37% per day per hour, but for all hourly time slots, it was below 10%. Therefore, the low-demand Finkenwerder station of the South area would not be a candidate for the introduction of a CEP strategy, as well as the Veddel and Wilhelmsburg stations of the South area, with a 12.13% and 15.47% average workload per day hour, respectively. The Süderelbe and Harburg stations would be poor candidates for the CEP strategy, with an average workload per day hour between about 22.35% and 27.72%, respectively.

To summarize, CEP strategy #1 [a] would waste resources by equally distributing one CEP to each of the stations, especially those in the South area. The four high-demand stations, especially stations in the East area (Barmbek, Sasel and Wandsbek) and the Stelling station in the West area, were clear candidates for the introduction of CEP strategies #1 [b, c]. However, during several time slots (e.g., morning slots), the average workload per hour was above 85% but remained below 100% only at the Stellingen and Wandsbek stations in the dataset for 2019. This could be especially critical in weeks 48 to 51 of the year, during the Advent season, as the average number of operations during these weeks was 19% to 28% higher than the weekly average of operations, potentially pushing workloads over 100%, especially during high-demand time slots in the morning. In the dataset for 2021, CEP strategy #1 [c] would be insufficient for the four high-demand stations during several time slots in the morning, with capacity even exceeding 100% during three times slots at the Sasel station. Thus, policymakers should consider a second CEP to cover critical high-demand time slots, using appropriate shifts similar to CEP strategies #2 [b, c], as illustrated in the next Section 3.2.2.

#### 3.2.2. CEP Strategy #2

CEP strategy #2 [a] would be similar to CEP strategy #1 [a] (equal distribution of one 24 h CEP to each of the 17 stations without considering their different demand patterns, as illustrated in Section 3.1.1) but assign a second 12 h CEP to the four high-demand stations (Barmbek, Sasel, Stellingen, and Wandsbek) so that the average workload per hour would mostly be below the critical 85% for all time slots [7]. Thus, policymakers would assign one CEP to each of the 17 stations for 24 h for 365 days, which would amount to **17 × 24 × 365 = 148,920 staff hours per year**. Then, the demand for a second 12 h CEP to the four high-demand stations (Barmbek, Sasel, Stellingen and Wandsbek) would have to be added for an appropriate shift from 9.00 to 19.00 to cover most of the peak hours to account for the findings in the post-COVID-19 datasets 2021 [7] and 2022 (**4 × 12 × 365 = 17,520 staff hours per year**). In total, **166,440 staff hours per year** would be needed.

However, again, similar to CEP strategy #1 [b], policymakers might only decide to introduce this strategy #2 [a] in the four high-demand stations (Barmbek, Sasel, Stellingen and Wandsbek) and in the two intermediate-demand stations (Altona and Bergedorf), amounting to **6 × 24 × 365 × 24 + 4 × 12 × 365 = 70,080 staff hours per year** for this strategy #2 [b]. Therefore, eleven 24 h CEPs could be saved compared to strategy #2 [a]. CEP strategy #2 [c] would then even save further costs by only implementing the strategy in the four high-demand stations (Barmbek, Sasel, Stellingen and Wandsbek): **4 × 24 × 365 × 24 + 4 × 12 × 365 = 52,560 staff hours**.

#### 3.2.3. CEP Strategy #3

The CEP strategies #3 [a, b] account for synergy effects to increase efficiency and effectiveness, with a lower number of total staff hours needed compared to strategies #1 [a, b, c] and #2 [a, b, c], but would induce a higher workload and stress for the CEPs. In CEP strategy #3 [a], low-demand stations would share resources (one 24 h CEP) and high-demand stations would obtain their own 24 h CEP, but some time slots might have high or uncovered demand, especially in the COVID-19 years [7]. In addition, a second shared or non-shared 12 h CEP would be assigned to high-demand stations in CEP strategy #3 [b] to release high-workload time slots. We proposed the following ten groupings of the 17 stations by accounting for neighborhood and appropriate shift times for CEP strategies #3 [a, b]:

- Group #1: Stations Süderelbe and Finkenwerder (South area):
  - one shared CEP for 24 h between these stations [a, b]
- Group #2: Stations Harburg, Veddel and Wilhelmsburg (South area):
  - one shared CEP for 24 h among these stations [a, b]
- Group #3: Stations Billstedt and Bergedorf (East area):
  - one shared CEP for 24 h between these stations [a, b]
- Group #4: Stations Innenstadt and Berliner Tor (East area):
  - one shared CEP for 24 h between these stations [a, b]
- Group #5: Stations Altona and Osdorf (West area):
  - one shared CEP for 24 h between these stations [a, b]
- Group #6: Station Rotherbaum (East area):
  - one CEP for 24 h for this station alone
- Group #7: Stations Stellingen and Alsterdorf (West area):
  - one shared CEP for 24 h between these stations [a, b]
  - one second shared CEP from 7.00–19.00 between these stations [b]
- Groups #8, #9, and #10: Station Barmbek, Sasel and Wandsbek (East area):
  - one CEP for 24 h for each of the groups [a, b]
  - one second non-shared CEP from 7.00–19.00 for each of the groups [b]

We approximated a minimum average hourly workload per CEP by summarizing the average hourly workloads of the stations assigned to a group, depending on the assignment and floating strategy. Such a strategy could be assigning a CEP to the more frequent station and covering other less frequent station(s) or assigning him/her to the nearest station after an operation However, by accounting for an average mission time of 60 min, used from a pilot study with larger catchment areas [8], our approximated results would provide a first insight for policymakers to choose appropriate candidate strategies for a pilot study or a simulation study. Then, policymakers should investigate different allocation and floating strategies of shared CEPs such as (1) the station to which they are assigned and (2) the station to which they have to return after an operation or at the end of their shift. Therefore, insights from reviews on ambulance emergency management studies might be essential for designing suitable allocation and floating strategies for CEPs [51,52,53,54,55,56].

When pooling stations, policymakers have to consider that the average hourly workload per CEP might have a realistic buffer up to 100%. Therefore, policymakers use a realistic workload limit of 85%, which is considered in Table 7. For example, in strategy #3 [a], the pooling the stations of group #5 (Altona and Osdorf) might not be possible due to the high average hourly workload of 78.29% plus the fact that ten hourly time slots would result in a workload above 85% and below 100% in 2019, while in strategy #3 [b], this group #5 would obtain a second 12 h CEP, resulting in a lower minimum hourly workload per CEP of about 55.09%. The same is true for group #7 (Stellingen and Alsterdorf). The other three high-demand stations from groups #8 (Barmbek), #9 (Sasel) and #10 (Wandsbek), as well as group #2 (Harburg, Veddel and Wilhelmsburg), might not urgently require a second CEP in 2019 because the minimum average hourly workload per CEP was below 70% for these groups, and no more than one hourly time slot was above 85%. However, as the demand was rising, as seen in the COVID-19 datasets for 2021 and 2022, additional resources would then be needed for them too.

From 2021 onwards, the second 12 h CEP plays an important role in strategy #3 [b], because the average hourly workload per CEP might then be frequently above the 85% without that additional resource, as illustrated in Table 7 (new based on [7]). Group #1 (Süderelbe and Finkenwerder) and group #6 (Rotherbaum) would not be assigned a second 12 h CEP. For groups #2, #8, #9 and #10, the additional resource was essential, and the demand of all hourly time slots for 2021 could be met, while for groups #3 and #4, only one hourly time slot in the morning would have a workload above 85%. However, for group #7 (Stellingen and Alsterdorf), the second 12 h CEP is highly essential, resulting in a lower average hourly workload per CEP of about 52.08% in strategy #3 [b] compared to 76.02% in strategy #3 [a]. The same applies to group #5 (stations Altona and Osdorf). Both groups #5 and #7 might need the second CEP, not only for 12 h but for three and five more hours after 19.00, respectively, to cope with the increased demand.

CEP strategy #3 [a] would require a total of only ten 24 h CEPs instead of 17 compared to CEP strategies #1 [a] and #2 [a], amounting to **10 × 24 × 365 = 87,600 staff hours per year,** but with a high workload for groups #5 and #7. For example, in group #7, ten hourly time slots would have a workload over 85% and two hourly time slots over 100% in 2019. To meet the increased demand for 2021 onwards and also the higher demand of weeks 47/48 to 51, all groups would require at least a second 12 h CEP, except groups #1 and #6, as illustrated by strategy #3 [b]. Groups #5 and #7 might need the second 12 h CEP for a few hours longer, or policymakers could consider not pooling these groups. Strategy #3 [b] would require eight 12 h CEPs (**8 × 12 × 365 = 35,040 staff hours per year**) compared to strategy #3 [a] amounting to **87,500 + 35,040 = 122,640 staff hours per year** in total. Policymakers could expand the working hours of the second CEP in groups #3 (one hour), #4 (one hour), #5 (three hours) and #7 (five hours) to meet the demand of all time slots, which would require **126,290 staff hours per year in total**.

## 4. Discussion

Our findings align with prior research in other countries where CEPs have successfully decreased non-urgent transport [10,12,13,16,17,19,20,21,22]. However, due to differing regulations and autonomy regarding CEPs in different countries, a direct comparison is difficult. For example, in a Finnish study [12], CEP deployment resulted in over 40% of patients avoiding hospital transport compared to approximately 20% in our 2019 dataset. However, it should be noted that, in contrast to the Canadian CEP model [13], which grants paramedics greater autonomy, German regulations currently limit the scope of CEPs to specific interventions. This may potentially mitigate the immediate impact on the demand for emergency medical services. Our study underscores the significant potential for CEPs and highlights the regulatory constraints that policymakers should address to facilitate the full integration of CEPs within Hamburg’s ARS.

By conducting statistical analyses for non-time-critical operations (approximately 16.4% in 2019), we investigated variations in demand patterns across the 17 stations located in three areas (East, South and West). Our findings revealed differences in the number of non-critical operations among these areas and within them. For policymakers, the high-demand stations in the East and West areas (Stellingen, Barmbek, Sasel and Wandsbek), followed by the higher-demand Altona (West area) and Bergedorf (East area) stations, are the best candidates for a CEP concept. Significant statistical differences were also reported for holidays in the weekly demand patterns. The time-of-day effects are a crucial consideration for effective resource allocation among stations, especially during peak hours (9:00–19:00). Based on these first and limited findings, we have proposed three viable, implementable resource allocation strategies for a CEP concept in Hamburg, contingent on the budget coverage and pooling strategies illustrated in Table 8 (new based on [7]).

The range of the workload among the stations varied (see Table 6), especially when assigning one 24 h CEP to low-demand stations, especially those in the south, such as Finkenwerder, with an average hourly workload of less than 5% (strategies #1 [a] and 2 [a]). Consequently, policymakers may consider implementing strategies #1 [b, c] and strategies #2 [b, c] to reduce costs by assigning one 24 h CEP to only the six higher-demand stations (Altona, Barmbek, Bergedorf, Sasel, Stellingen and Wandsbek) or only to the four high-demand stations (Barmbek, Sasel, Stellingen and Wandsbek), respectively. However, strategies #1 [a, b, c] would not be sufficient to meet increasing demand in the years 2021 onwards for the four high-demand stations of Barmbek, Sasel, Stellingen and Wandsbek, as illustrated in Table 7 and Table 8. By assigning a second 12 h CEP from 9.00 to 19.00 to the four high-demand stations (Barmbek, Sasel, Stellingen and Wandsbek) in strategies #2 [a, b, c], demand could be met in all stations in the year 2021. In terms of coverage and CEP resources needed, strategies #3 [a, b] would be the most promising. These strategies would see low-demand stations grouped together, with ten 24 h CEPs required to account for synergy effects.

Depending on the budget and coverage strategy, policymakers would select one of the above options. For tight budgets, strategies #1 [b, c] and strategies #2 [b, c] would be an option to introduce the CEP strategy in only the six higher-demand stations (about 35% area coverage rate) or even in just the four high-demand stations (23.5% area coverage rate), respectively. However, to ensure the feasibility of the CEP strategy for high-demand years, such as 2021, strategies #1 [a, b, c] might not be sufficient, as the high-demand stations would then require a second CEP for 12 h, as in strategies #2 [a, b, c]. If geographical pooling of stations is a viable option, strategies #3 [a, b] would be the most favorable to fully cover the City of Hamburg with 17 stations (100% area coverage rate). To be feasible for a higher-demand year such as 2021, only strategy #3 [b] would be viable with the consideration of providing a second CEP to four groups for more than 12 h, which would then require 126,290 staff hours per year.

In general, the increasing demand for the ARS is partly due to the treatment of patients outside of business hours in the context of contractual medical care [24], which is not the main intention of an ARS. The solution to this access problem lies in the extension of the opening hours of contractual medical care providers, especially the concept of primary healthcare centers, improving emergency hotlines and introducing community nurses. For instance, Seeger et al. [25] found that, in a survey of over 5000 German patients, many patients attempted to contact the rescue services, despite these services not being the intended purpose of the ARS. The CEP system also integrates well with modern app-based first-responder alert systems and publicly located automated external defibrillators, further reducing response times for first aid [26]. The final aim is mobile-integrated healthcare, with patient-centered coordination of physician-led teams that include essential healthcare professionals (e.g., CEPs, community nurses, social/mental healthcare workers, family doctors, other medical specialists) [27]. Furthermore, the hospital sector of the City of Hamburg needs to adapt by improving the handling and treatment of emergency patients, as well as enlarging the related necessary capacities.

In 2023, the ambulance rescue system’s policymakers in Hamburg decided against adopting the CEP strategy due to the high costs and efforts of changing the current system, despite the presentation of the initial results of our promising study. Instead, they opted to procure ten additional ambulance vehicles to address the rising demand. These vehicles are primarily operated by relief organizations such as the Arbeiter-Samariter-Bund, Johanniter and the German Red Cross, which play a vital role in meeting the increasing demand of the City of Hamburg.

However, should the diffusion of successful paramedic strategies in Germany continue to progress, and/or should the healthcare coverage situation worsen, it is possible that these policymakers might change their position in the future. In that case, our preliminary, albeit limited, findings could form the basis for a subsequent, more extensive pilot or simulation study. Such a study could utilize recent post-COVID-19 data due to the increased demand for services across the entire emergency rescue system and incorporate a broader cost-effectiveness analysis for the City of Hamburg, Germany. Some of the increase in demand is caused by the fact that COVID-19 has become prevalent and long COVID-19 effects have manifested in the population as well as in the workforce, which might increase the demand for ambulance services, and thus, CEPs [41,42,43].

## 5. Conclusions

For healthcare policymakers, CEP units (one parametric on a basic vehicle) play a crucial role in enhancing first-responder networks for non-critical patients worldwide. This is achieved by reducing response time and conserving vital ambulance resources. It supports the concepts of community nurses for home-based care, medical emergency hot-lines, medical doctors in private offices and emergency departments in hospitals. The CEP units also integrate well with modern app-based first-responder alert systems and publicly located automated external defibrillators, further reducing response times for first aid. This is why such a CEP approach presents a viable solution to address the growing demand for ambulance healthcare services provided by the fire brigade in the City of Hamburg, Germany. To address the research question of how the number of non-time-critical operations varies across different city areas and which time-related staffing strategies might be most beneficial for a certain area or the city, a demand and supply analysis was conducted using statistical methods. Three viable, implementable resource allocation strategies for a CEP concept in Hamburg have been proposed, which could form the basis for a subsequent pilot or simulation study, contingent on budget, coverage and pooling strategies.

Whilst the study’s findings offer valuable insights into potential CEP deployment in Hamburg, there remain several limitations that should be noted. Firstly, the data reflect only 2019 (pre-pandemic) and 2021 (during the pandemic) operations, which may not fully capture post-pandemic changes in ambulance demand. Secondly, we assumed an average of 60 min per CEP call based on the existing literature, but real operation times could vary significantly. Thirdly, our analysis used aggregated station-level data, which might overlook local socioeconomic or demographic nuances. Fourthly, no comprehensive cost-effectiveness comparison was performed between the CEP concept and the concept of increasing conventional ambulance staff and vehicles to meet the increasing demand. Finally, the entire emergency healthcare, rescue and treatment system of the City of Hamburg could be incorporated and examined due to the increasing demand for emergency services. Future research should address these issues by employing more granular, up-to-date data and broader economic evaluations.

## Figures and Tables

**Table 1:** Variables for the statistical models.

Variable	Type	Number [Range]
**Stations**	Categorial	17 [1 = Altona,…, 17 = Wandsbek]
**Months**	Ordinal	12 [1 = January,…, 12 = December]
**Weeks**	Ordinal	52 [1…52]
**Day of the Year**	Numeric [Time Series]	365 [*t*_0_ = 1 January, …, *t*_364_ = 31 December]
**Days of the Week**	Ordinal	1 [1 = Monday,…, 7 = Saturday]
**Hours of the Day**	Ordinal	24 [1 = “0.00–0.59”,…, 24 = “23.00–23.59”]
**Breaks**	Binary	[1 = true, 0 = false]
**Events**	Binary	[1 = true, 0 = false]
**Holidays**	Binary	[1 = true, 0 = false]

**Table 2:** The size and population density of the 17 major ARS stations in the City of Hamburg, Germany, 2019.

Station	Abbrevation Station	Area	Population Size	Area Size in km^2^	Population Density Per km^2^
**Altona**	Alo	West	70,791	11.42	6199.41
**Alsterdorf**	Als	West	152,952	41.88	3652.06
**Barmbek**	Bar	East	170,752	25.265	6761.12
**Bergedorf**	Beg	East	114,212	129.25	883.68
**Berliner Tor**	Bet	East	78,123	13.37	5841.85
**Billstedt**	Bil	East	56,125	57.38	978.19
**Finkenwerder**	Fin	South	12,846	56.18	228.64
**Harburg**	Har	South	109,213	47.08	2319.73
**Innenstadt**	Inn	West	21,607	6.87	3145.58
**Osdorf**	Osd	West	204,793	57.69	3550.01
**Rotherbaum**	Rot	West	109,294	11.01	9928.60
**Sasel**	Sas	East	169,192	86.42	1957.72
**Stellingen**	Ste	West	272,006	49.08	5542.67
**Süderelbe**	Sue	South	58,611	59.26	989.07
**Veddel**	Ved	South	22,271	24.72	901.11
**Wandsbek**	Wan	East	123,594	45.39	2722.76
**Wilhelmsburg**	Wil	South	51,689	26.60	1942.90
**Average in East Area**		East	51,689	47.08	1276.29
**Average in South Area**		South	118,666	59.51	3190.87
**Average in West Area**		West	138,574	29.66	5336.39
**Average per Station**			105,769	44.05	3385.00
**Hamburg Area (Total)**			**1,798,071**	**748.85**	**2401.10**

**Table 3:** Number of time-critical and non-time-critical operations performed by the 17 major ARS stations in the City of Hamburg, Germany, 2019.

Station	Area	Number of Non-Time-Critical Operations				
		Total Number	With Transportation to Hospital		Without Transportation to Hospital	
			Number	%	Number	%
**Altona**	West	3877	3322	85.68%	555	16.71%
**Alsterdorf**	West	2168	1752	80.81%	416	23.74%
**Barmbek**	**East**	**4749**	**3927**	**82.69%**	**822**	**20.93%**
**Bergedorf**	East	3426	2699	78.78%	727	26.94%
**Berliner Tor**	East	2823	2369	83.92%	454	19.16%
**Billstedt**	East	1973	1519	76.99%	454	29.89%
***Finkenwerder***	*South*	*383*	*314*	*81.98%*	*69*	*21.97%*
**Harburg**	South	2431	2076	85.40%	355	17.10%
**Innenstadt**	West	2541	2205	86.78%	336	15.24%
**Osdorf**	West	2982	2557	85.75%	425	16.62%
**Rotherbaum**	West	2086	1784	85.52%	302	16.93%
**Sasel**	**East**	**4842**	**4203**	**86.80%**	**639**	**15.20%**
**Stellingen**	**West**	**4494**	**3674**	**81.75%**	**820**	**22.32%**
**Süderelbe**	South	1959	1669	85.20%	290	17.38%
**Veddel**	South	1063	910	85.61%	153	16.81%
**Wandsbek**	**East**	**4883**	**3967**	**81.24%**	**916**	**23.09%**
**Wilhelmsburg**	South	1355	1140	84.13%	215	18.86%
**Average per Station**		**2825.59**	**2358.06**	**83.47%**	**467.53**	**19.93%**
**Average in East Area**		22,696	18,684	82.32%	4012	17.68%
**Average in South Area**		7191	6109	84.95%	1082	15.05%
**Average in West Area**		18,148	15,294	84.27%	2854	15.73%
**Total**	**292,856 (100%)**	**48,035 (16.40%)**	**40,087 (13.68%)**	**100%**	**7948 (2.71%)**	**100%**

**Table 4:** Number of non-time-critical operations performed on weekdays by the 17 major ARS stations in the City of Hamburg, Germany, 2019.

Weekday	Number of Non-Time-Critical Operations	Percentage of Non-Time-Critical Operations
**Monday**	7125	14.83%
**Tuesday**	6959	14.49%
**Wednesday**	6599	13.74%
**Thursday**	6833	14.23%
**Friday**	6812	14.18%
**Saturday**	6973	14.52%
**Sunday**	6734	14.02%
**Average per day**	**6862.14**	**14.29%**
**Total**	**48.035**	**100.00%**

**Table 5:** Wandsbek station, 2019: workload calculation for the CEP strategy #1.

Hourly Time Slot	Average Rate of Non-Time-Critical Operations	Operation Time (in Minutes)	Average Demand (in Minutes)	Number of Community Paramedics	Supply (in Minutes)	Average Workload [Demand/Supply] (%)
**00.00–00.59**	0.3534	60	21.21	1	60	35.34%
**01.00–01.59**	0.3370	60	20.21	1	60	33.70%
**02.00–02.59**	0.3233	60	19.40	1	60	32.33%
**03.00–03.59**	0.2301	60	13.81	1	60	23.01%
**04.00–04.59**	0.2767	60	16.60	1	60	27.67%
**05.00–05.59**	0.2356	60	14.14	1	60	23.56%
**06.00–06.59**	0.3452	60	20.71	1	60	34.52%
**07.00–07.59**	0.4740	60	28.44	1	60	47.40%
**08.00–08.59**	0.6548	60	39.29	1	60	65.48%
**09.00–09.59**	0.7562	60	45.37	1	60	75.62%
**10.00–10.59**	**0.8877**	**60**	**53.26**	**1**	**60**	88.77% (*)
**11.00–11.59**	0.8301	60	49.81	1	60	83.01%
**12.00–12.59**	0.7616	60	45.70	1	60	76.16%
**13.00–13.59**	0.7397	60	44.38	1	60	73.97%
**14.00–14.59**	0.7178	60	43.07	1	60	71.78%
**15.00–15.59**	0.6110	60	36.66	1	60	61.10%
**16.00–16.59**	0.7699	60	46.19	1	60	76.99%
**17.00–17.59**	0.7178	60	43.07	1	60	71.78%
**18.00–18.59**	0.6247	60	37.48	1	60	62.47%
**19.00–19.59**	0.6575	60	39.45	1	60	65.75%
**20.00–20.59**	0.5945	60	35.67	1	60	59.45%
**21.00–21.59**	0.5534	60	33.21	1	60	55.34%
**22.00–22.59**	0.4466	60	26.80	1	60	44.66%
**23.00–23.59**	0.4685	60	28.11	1	60	46.85%
**Total**	**0.5569** **(average)**	**1400** **(sum)**		**1**	**1440** **(sum)**	**55.70%** **(average)**

* Above the required 85% limit.

**Table 6:** Average hourly workload of the 17 stations and the related feasibility of strategy #1 in the years 2019 and 2021.

Station	Average Hourly Workload Strategy #1 in 2019	Feasibility 2019	Feasibility 2021
**Altona [b]**	44.26%	sufficient	sufficient
**Alsterdorf [a]**	24.75%	sufficient	sufficient
**Barmbek [b, c]**	54.19%	sufficient	6 time slots over 85% and 1 time slot over 100%
**Bergedorf [b]**	39.09%	sufficient	sufficient
**Berliner Tor [a]**	32.23%	sufficient	sufficient
**Billstedt [a]**	22.51%	sufficient	sufficient
**Finkenwerder [a]**	4.37%	sufficient	sufficient
**Harburg [a]**	27.73%	sufficient	sufficient
**Innenstadt [a]**	28.98%	sufficient	sufficient
**Osdorf [a]**	34.03%	sufficient	sufficient
**Rotherbaum [a]**	23.79%	sufficient	sufficient
**Sasel [b, c]**	55.26%	1 time slot over 85%	1 time slot over 85% and 3 time slots over 100%
**Stellingen [b, c]**	51.27%	sufficient	3 time slots over 85%
**Süderelbe [a]**	22.35%	sufficient	sufficient
**Veddel [a]**	12.13%	sufficient	sufficient
Wandsbek [b, c] *	55.70%	1 time slot over 85%	6 time slots over 85% and 2 time slots over 100%
**Wilhelmsburg [b, c]**	15.47%	sufficient	sufficient

* As an example, the feasibility table for 2019 for the Wandsbek station is presented in Table 5.

**Table 7:** Average hourly workload of the 17 stations and the related feasibility of strategy #3 in the years 2019 and 2021.

Groups	Average Hourly Workload Strategy #3 [a] (Feasibility in 2019 and 2021)	Average Hourly Workload Strategy #3 [b] (Feasibility in 2019 and 2021)
**(** **1) Süderelbe and Finkenwerder**	26.72%	-
**(** **2) Harburg, Veddel and Wilhelmsburg**	55.33% (2019: 1 > 85%; 2021: 3 > 85%, 1 > 100%)	38.08%
**(** **3) Billstedt and Bergedorf**	61.60% (2021: 8 > 85%; 2 > 100%)	40.45% (2021: 1 > 85%)
**(** **4) Innenstadt and Berliner Tor**	61.21% (2021: 10 > 85%)	43.83% (2021: 1 > 85%)
**(** **5) Altona and Osdorf**	78.29% (2019: 10 > 85%, 2 > 100%; 2021: 3 > 85%, 13 > 100%)	55.09% (2019: 1 > 85; 2021: 3 >85%; 2 > 100%)
**(** **6) Rotherbaum**	23.79%	-
**(** **7) Stellingen and Alsterdorf**	76.02% (2019: 11 > 85%; 2021: 3 > 85%,12 > 100;)	52.08% (2019: 1; 2021: 2 > 85%; 1 > 100%)
**(** **8) Barmbek**	54.19% (2021: 6 > 85%; 1 > 100%)	36.55%
**(** **9) Sasel**	55.26% (2019: 1 > 85%; 2021: 3 > 85%; 3 > 100%)	37.28%
**(** **10) Wandsbek**	55.70% (2019: 1 > 85%; 2021: 6 > 85%; 2 > 100%)	37.89%

**Table 8:** Comparison of the CEP strategies regarding coverage, resource utilization and feasibility for the City of Hamburg, Germany in the year 2021.

CEP Strategy	Number of 24 h Community Paramedics (Area Coverage Rate)	Number of 12 h Community Paramedics	Number of Staff Hours Per Day	Number of Staff Hours Per Year *	Feasibility in 2021
**Strategy #1 [a]: one 24 h CEP** **for each station**	17 (100%)	0	408	148,920	four high-demand stations
**Strategy #1 [b]: one 24 h CEP** **for the six higher-demand stations**	6 (35.3%)	0	144	52,560	four high-demand stations
**Strategy #1 [c]: one 24 h CEP** **for the four high-demand stations**	4 (23.5%)	0	96	35,040	four high-demand stations
**Strategy #2 [a]: one 24 h CEP** **for each station and** **a second 12 h one for four higher-demand stations**	17 (100%)	4	456	166,440	
**Strategy #2 [b]: one 24 h CEP** **for six higher-demand stations and** **a second 12 h one for the four high-demand stations**	6 (35.3%)	4	192	70,080	
**Strategy #2 [c]: one 24 h CEP** **for four high-demand stations and** **a second 12 h one for the four high-demand stations**	4 (23.5%)	4	144	52,560	
**Strategy #3 [a]: one shared 24 h CEP for each group (ten groups including 17 stations)**	10 (100%)	0	240	87,600	high feasibility for eight groups
**Strategy #3[b]: one shared 24 h CEP for each group and** **a second 12 h one for the eight higher-demand groups (ten groups including 17 stations)**	10 (100%)	8	336	122,640	Slight feasibility for two groups and intermediate feasibility for two groups

* Calculation: number of staff hours per day∗365.

## Data Availability

The main data that support the findings of this study are available in Swyter [Number]. Further data are restricted by the ambulance rescue organization and are therefore not publicly available.
